# The need for widely available genomic testing in rare eye diseases: an ERN-EYE position statement

**DOI:** 10.1186/s13023-021-01756-x

**Published:** 2021-03-20

**Authors:** Graeme C. Black, Panagiotis Sergouniotis, Andrea Sodi, Bart P. Leroy, Caroline Van Cauwenbergh, Petra Liskova, Karen Grønskov, Artur Klett, Susanne Kohl, Gita Taurina, Marius Sukys, Lonneke Haer-Wigman, Katarzyna Nowomiejska, João Pedro Marques, Dorothée Leroux, Frans P. M. Cremers, Elfride De Baere, Hélène Dollfus, ERN-EYE study group, Gavin Arno, Jane Ashworth, Isabelle Audo, Giacomo Bacci, Vilma Jurate Balciuniene, Sara Bargiacchi, Mette Bertelsen, Graeme Black, Camiel Boon, Dominique Bremond-Gignac, Luca Buzzonetti, Patrick Calvas, Adela Chirita-Emandi, Davit Chokoshvili, Frans Cremers, Avril Daly, Hélène Dollfus, Susan Downes, Adriano Fasolo, Christina Fasser, Dominik Fischer, Pina Fortunato, Arvydas Gelzinis, Karen Grønskov, Lonneke Haer-Wigman, Georgina Hall, Steffen Hamann, Elise Héon, Giancarlo Iarossi, Caroline Iberg, Gaëlle Jouanjan, Helena Kaariainen, Kamron Kahn, David Keegan, Artur Klett, Susanne Kohl, Michael Laengsfeld, Alberta Leon, Dorothée Leroux, Bart Peter Leroy, Petra Liskova, Birgit Lorenz, Riccardo Maggi, Joao Pedro Marques, Laura Mauring, Paolo Melico, Isabelle Meunier, Saddek Mohand-Saïd, Cristina Monterosso, Paola Morandi, Katarzyna Nowomiejska, Francesco Parmeggiani, Ilaria Passerini, Valérie Pelletier, Francesca Peluso, Yaumara Perdomo, Emilio Rapizzi, Laura Roos, Susanne Roosing, Jean-Michel Rozet, Panos Sergouniotis, Francesca Simonelli, Andrea Sodi, Jane Sowden, Katarina Stingl, Dominique Sturz, Marius Sukys, Agnese Suppiej, Gita Taurina, Francesco Testa, Anna Tracewska, Giovanna Traficante, Sandra Valeina, Caroline Van Cauwenbergh, Elfride De Baere, Russell Wheeler, Thomas Wheeler-Schilling, Patrick Yu-Wai-Man, Christina Zeitz, Reda Žemaitienė

**Affiliations:** 1 Manchester Centre for Genomic Medicine, Saint Mary’s Hospital and Manchester Royal Eye Hospital, Manchester Academic Health Science Centre, Manchester University Hospitals NHS Foundation Trust, Manchester, UK; 2 Azienda Ospedaliero Universitaria Careggi, Firenze, Italy; 3 Department of Ophthalmology, Ghent University Hospital, Ghent, Belgium; 4 Department of Head and Skin, Ghent University, Ghent, Belgium; 5 Center for Medical Genetics Ghent, Ghent University Hospital, Ghent, Belgium; 6 Division of Ophthalmology and Center for Cellular and Molecular Therapeutics, The Children’s Hospital of Philadelphia, Philadelphia, PA USA; 7 Department of Ophthalmology, General University Hospital in Prague, Prague, Czech Republic; 8 Rigshospitalet, Glostrup, Denmark; 9 East Tallinn Central Hospital, Tallinn, Estonia; 10 Institute for Ophthalmic Research, Centre for Ophthalmology, University Hospital Tübingen, Tübingen, Germany; 11 Children’s Clinical University Hospital, Riga, Latvia; 12 Hospital of Lithuanian, University of Health Science, Kauno Klinikos, Lithuania; 13 Department of Human Genetics, Radboudumc, Nijmegen, Netherlands; 14 Department of General Ophthalmology, Medical University, Lublin, Poland; 15 Centro Hospitalar E Universitário de Coimbra (CHUC), Coimbra, Portugal; 16 ERN-EYE Coordination Center, Hopitaux Universitaires de Strasbourg, Strasbourg, France; 17 Center for Medical Genetics, Department of Biomolecular Medicine, Ghent University and Ghent University Hospital, Ghent, Belgium; 18 CARGO, Hôpitaux Universitaires de Strasbourg, Strasbourg, France; 19 U-1112, Inserm, Faculté de Médecine, Université de Strasbourg, Strasbourg, France

**Keywords:** Genetic and genomic testing, Rare eye diseases, ERN-EYE, Position statement

## Abstract

**Background:**

Rare Eye Diseases (RED) are the leading cause of visual impairment and blindness for children and young adults in Europe. This heterogeneous group of conditions includes over 900 disorders ranging from relatively prevalent disorders such as retinitis pigmentosa to very rare entities such as developmental eye anomalies. A significant number of patients with RED have an underlying genetic etiology. One of the aims of the European Reference Network for Rare Eye Diseases (ERN–EYE) is to facilitate improvement in diagnosis of RED in European member states.

**Main body:**

Technological advances have allowed genetic and genomic testing for RED. The outcome of genetic testing allows better understanding of the condition and allows reproductive and therapeutic options. The increase of the number of clinical trials for RED has provided urgency for genetic testing in RED. A survey of countries participating in ERN-EYE demonstrated that the majority are able to access some forms of genomic testing. However, there is significant variability, particularly regarding testing as part of clinical service. Some countries have a well-delineated rare disease pathway and have a national plan for rare diseases combined or not with a national plan for genomics in medicine. In other countries, there is a well-established organization of genetic centres that offer reimbursed genomic testing of RED and other rare diseases. Clinicians often rely upon research-funded laboratories or private companies. Notably, some member states rely on cross-border testing by way of an academic research project. Consequently, many clinicians are either unable to access testing or are confronted with long turnaround times. Overall, while the cost of sequencing has dropped, the cumulative cost of a genomic testing service for populations remains considerable. Importantly, the majority of countries reported healthcare budgets that limit testing.

**Short conclusion:**

Despite technological advances, critical gaps in genomic testing remain in Europe, especially in smaller countries where no formal genomic testing pathways exist. Even within larger countries, the existing arrangements are insufficient to meet the demand and to ensure access. ERN-EYE promotes access to genetic testing in RED and emphasizes the clinical need and relevance of genetic testing in RED.

## Background

Technological advances have allowed genetic and genomic testing for Rare Eye Diseases (RED). The outcome of genetic testing allows better understanding of RED and allows reproductive and therapeutic options. Despite these advances critical gaps in testing remain in European member states, especially in smaller countries. Even within larger countries, the existing arrangements are insufficient to meet the demand and to ensure equity of access. The European Reference Network (ERN) initiative, a cross-border cooperation between healthcare providers and researchers from across the European Union, has been created to improve diagnosis and treatment of complex or rare medical conditions that require specialised treatment, knowledge and resources [1]. ERN-EYE promotes access to genetic testing in RED and emphasizes the clinical need and relevance of genetic testing in RED.

## Main text

There are 24 thematic ERNs, including ERN-EYE, whose focus is on RED [2]. The ERNs seek to aggregate healthcare providers in order to improve patient access to healthcare information and thereby increase treatment options. They aim to do this by (i) creating innovative care models, in particular using digital technologies, (ii) enhancing research through the increase of the size and power of clinical studies as well as epidemiological registries and (iii) enabling sharing of costly resources which ultimately leads to more sustainable national healthcare systems. The overarching objective is to improve health outcomes for the large numbers of patients in the EU suffering from rare and often complex conditions.

RED are the leading cause of visual impairment and blindness for children and young adults in Europe [3, 4]. This heterogeneous group of conditions includes over 900 disorders ranging from relatively prevalent disorders such as retinitis pigmentosa (estimated prevalence of 1 in 4,000) to very rare entities described only once or twice in medical literature [5]. ERN-EYE is structured around 4 clinical thematic working groups (Retina, Neuro-ophthalmology, Paediatric, Anterior segment) and 6 transversal working groups (Low vision, Genetic diagnostic, Registries, Research, Education / Training, Communication) [2]. Notably, the ERN-EYE has organised workshops on diverse areas ranging from clinical terminology standardisation (Mont Sainte-Odile workshop, 2017) to genomic testing (Florence workshop, 2018) and clinical trials (Strasbourg workshop, 2019) [6].

The advance towards personalization of medicine is accelerating [7]. For rare diseases, including RED, there is now a general understanding that patients often experience delayed diagnosis, which in turn leads to poor access to appropriate treatment and management protocols. For RED, a significant number of patients have an underlying genetic etiology. Effective and individualized approaches to clinical management are consequently dependent upon a comprehensive means of delivering genetic or genomic testing [8]. Genomic testing allows a precise diagnosis of highly heterogeneous disorders, improves counselling (e.g. understanding prognosis; facilitating reproductive decision-making) and is increasingly important in directing treatment options [9].

### Genomic approaches can improve diagnosis and management of RED

There are now numerous examples demonstrating clinical benefit of genomic testing in RED. For example, for oculocutaneous albinism, genetic diagnostic approaches provide a positive diagnosis in over 75% of cases. This not only achieves a diagnosis in early life for individuals with reduced vision but also allows identification of syndromic forms including the 1 in 30 cases of apparently uncomplicated albinism that represent unsuspected cases of Hermansky-Pudlak syndrome implying specific surveillance and care [10].

Leber Congenital Amaurosis (LCA) is the earliest onset and most severe form of inherited retinal diseases (IRD) [11]. This group of conditions is caused by genetic alterations in over 20 genes and is also the field where most clinical research is performed to date [12–14]. Some examples are given where comprehensive genomic testing leads to a molecular diagnosis and offers therapeutic perspectives. A first example are pathogenic variants in the RPE-specific gene *RPE65* encoding a protein member of the visual cycle that regenerates retinal. The recent FDA and EMA approval of *voretigene neparvovec-rzyl* for the treatment of LCA patients with biallelic *RPE65* mutations, as a landmark of novel gene-directed therapy, paved the way for successful treatment [15–18]. A second example is a recurrent deep-intronic pathogenic variant in *CEP290*, a gene encoding a key component of the connecting cilium. There are promising clinical studies suggesting potential for intravitreally delivered antisense oligonucleotide (AON) therapy and for gene editing using CRISPR/Cas9 [19–21]. Pathogenic variants in *CEP290* and other cilia-related genes (e.g. *IQCB1*) can predispose for multi-systemic complications including renal failure [22, 23]. Other examples requiring an early diagnosis are *AILP1*- and *GUCY2D*-associated LCA given the ongoing therapeutic efforts [24–27].

Moreover, *CLN3*-associated Batten disease, first diagnosed by ophthalmologists, is another example where early diagnosis is critical to direct management, counseling, and support for young patients and their families. The systemic therapeutic options for this disease in early-phase clinical trial benefit from a start at the earliest stage of disease [28, 29].

Other examples are pathogenic variants identified in disease genes implicated in achromatopsia [30], choroideremia [31], Stargardt disease (STGD1), X-linked retinitis pigmentosa and other IRD [33, 34] that are eligible for the huge range of clinical trials being undertaken currently [12–14]. Specifically, rare and recurrent deep-intronic pathogenic variants (total: 355) in *ABCA4* associated with STGD1 in ~10% of cases allow the design of novel RNA splice modulation therapies using AONs [35–37].

Patient groups, clinicians and scientists together recognize an urgent need for widespread availability of genomic testing for RED to avoid the so-called ‘diagnostic odyssey’ - an extended and distressing period, often unsuccessful, characterised by multiple sequential investigations. By providing a definitive molecular diagnosis this can strongly facilitate clinical and personal decision-making [38, 39].

### What is the current picture of genomic testing in RED?

Adoption of genomic testing for RED has accelerated considerably over the past 10 years due to the availability of ‘next generation sequencing’ (NGS), a technological advance allowing massively parallel sequencing of multiple nucleic acid targets [38]. This technique is increasingly being deployed in the clinical diagnostic setting and it has allowed affordable analysis of complete genomes [40, 41].

A survey of countries participating in ERN-EYE demonstrated that the majority are able to access some forms of genomic testing. However, access is still far from universal and there is significant variability of delivery, particularly in the degree to which different countries are able to provide testing as part of clinical service. It is not uncommon for clinicians to have to rely partly or completely upon either research-funded laboratories (for example in the Czech Republic) or private companies. Notably, some member state relies mainly on cross-border testing either by way of an academic research project. For example, research-based sequencing of the entire *ABCA4* gene for variants associated with STGD1 in the Netherlands and Belgium has yielded bi-allelic variants in ~500 probands ascertained worldwide, including many undiagnosed families from Eastern European countries [35–37]. Currently 2,000 STGD1 and STGD-like maculopathy probands have been sequenced for mutations in *ABCA4* and *PRPH2,* solving ~50% of the cases.

In the US, *Invitae* has announced a free sequencing service for RED probands from the US based on a partnership with *Spark Therapeutics* [41]. The Foundation Fighting Blindness, in partnership with *Blueprint Genetics* and *InformedDNA*, offers free genetic testing and counselling to individuals living in the US or US territories and clinically diagnosed with an IRD [42].

In Europe, some countries have a very well delineated rare disease pathway (summarized in Table 1). In France for example, there is a long-standing national centralized organizational plan for rare diseases (*Plan National Maladies Rares*) [43] now combined with a centralized national plan for genomics in medicine *(Plan France Médecine Génomique)* [44]. In the UK, a small number of Genomic Laboratory Hubs and a highly productive national initiative (100,000 Genomes; Genomic England) allow relatively frictionless access to testing [45]. In Belgium and the Netherlands there is a well-established organization of genetic centres with good access to reimbursed genomic testing of RED and other rare diseases. In Germany, academic genetic centres, private genetic laboratories but also industrial laboratories offer this service. Other member states such as Italy rely on regional organisation where University centres have, over time, developed significant expertise in specific RED fields.

**Table 1:** Rare disease pathway summary and access to genetic testing by country

Questions to ERN-eye members? (October 2018 with March 2020 update)	BE	CZ	DK	ES	FR	GE	IT	LV	LT	NL	UK	PL	PT
Are there national initiatives for genetic testing (GT) such a National Plan?	Y	Y	N	Y	Y	N	N	Y	Y	N	Y	N	N
Is there a unique national model for the consent form?	N	N	Y	N	Y	N	N	Y	N	N	Y	N	N
(thus HCP specific)	Y	Y	Y	Y	Y	N	Y	N	Y	Y	Y	Y	Y
Are most of the GTs done with academic hospital laboratories?	Y	Y	N	Y	N	Y	N	Y	N	Y	N	Y	Y
Are GTs done by industrial partners?	N	Y	N	Y	N	N	Y	95%	rare	N	N	Y	Y
Are there samples sent abroad for GT?	Y	Y	Y	Y	Y	Y	Y	N	N	Y	rare	Y	Y
Can ophthalmologists prescribe the GTs?	Y	Y	N	Y	Y	Y	N	Y	Y	Y	Y	N	N
Are there national rules for the prescription of GTs?	Y	Y	Y	Y	Y	N	Y	Y	Y	Y	Y	Y	Y
Are there multidisciplinary meetings in the GT course?	Y	Y	Y	Y	Y	Y	Y	Y	Y	Y	Y	Y	Y
Do you have access to Sanger sequencing?	Y	Y	Y	Y	Y	Y	Y	Y	Y	Y	Y	Y	Y
Do you have access to panel-based testing?	Y	Y	Y	Y	Y	Y	Y	Y	Y	Y	Y	Y	Y
Do you have access to whole exome sequencing (WES) for GT in your country?	Y	N	N	Y	Y	Y	N	N	Y	Y	Y	N	N
Do you have access to whole genome sequencing (WGS) for GT in your country?	N	N	N	N	N	N	N	N	N	Y	N*	N	N
Do you have access to WES for research only?	Y	Y	Y	Y	N	N	Y	Y	N	Y	N	N	N
Do you have access to WGS for research only?	Y	Y	Y	Y	Y	Y	Y	Y	Y	Y	Y	N	Y
Is the patient reimbursed for GT (gene panels, Sanger)?	Y	Y	Y	Y	Y	Y	Y	Y	Y	Y	Y	N	Y
Is the patient reimbursed for WES?	Y	−na	−na	−na	Soon	Y	N	N	−na	Y	Y	N	N
Is the patient reimbursed for WGS?	N	−na	N	N	N	N	N	N	Y	N	N	N	N
Are there enough specialists in genetic ophthalmology in your MS?	Y	N	Y	Y	N	N	N	N	N	Y	Y	N	N
Is there a national genetic database?	N	N	N	Y	N	N	N	Y	Y	Y	N	N	N

*Due to be available from April 2021

Abbreviations used: BE: Belgium; CZ: Czech Republic; DK: Denmark; ES: Spain; FR: France; GE: Germany; IT: Italy; LV: Latvia; LT: Lithuania; NL: Netherlands; UK: United Kingdom; PL: Poland; PT: Portugal

Within this overall picture, critical gaps in testing remain, especially in a number of smaller countries where no formal genomic testing structures exist. Notably, even within larger countries, the existing arrangements are insufficient to meet the demand and to ensure equity of access. Consequently, across the EU there are large numbers of clinicians and affected families who are either unable to access testing or who have to wait for considerable periods of time to receive results. Overall, while the cost of genomic sequencing has dropped at an extraordinary rate over the past decade, the cumulative cost of providing a comprehensive genomic testing service for populations remains considerable. Importantly, the majority of EU countries reported healthcare budgets that limit testing despite the fact that increase in demand (i.e. numbers of patients requiring testing) is inevitable [46].

#### Clinical utility: making the argument to justify genomic testing

It is perhaps not surprising that translation of clinical, technological and research advances into routine healthcare is slow. Undoubtedly, the adoption of a clinically relevant intervention—in this case, genomic testing—is more likely where its ability to influence management and health outcomes has been clearly demonstrated. Therefore, a focus on clinical benefit (‘clinical utility’) of genomic testing remains an urgent requirement to provide a clear evidence for widespread implementation [47, 48]. To date, compiling such evidence for RED has been slow. However, evidence of clinical utility has been demonstrated for small groups of patients [49–53]. Additional, well-designed studies of broader scale are becoming available [7, 54].

#### Training and mainstreaming of genomic medicine

Genomic testing is only one of the barriers that exist for effective diagnosis and management for individuals with RED. It is clear that the number of healthcare professionals and genetic counsellors who specialise in ophthalmic genetics is another important limiting factor, even in settings where genomic testing is readily available. Notably, at present, care for families with RED is generally delivered by a few “super-specialists” in ophthalmic genetics who work within a relatively small number of academic centres. Given the cumulative prevalence and overall number of RED, and the increasing recognition of clinical need, this dependence of small groups of experts is likely to be unsustainable.

Broadening access to genomic testing will require an expansion of the group of clinicians who are willing and able to order such diagnostic tests. Since this requires specialist knowledge, training of a wider group of clinicians at all strata of seniority will be necessary. While in the longer term this sits within medical schools and professional curricula, in the shorter term it will be critical to provide professional development that enables up-skilling of existing clinical workforces. There will be different levels of skills required for different groups of clinicians. Paediatric ophthalmologists and medical retina specialists who encounter RED more frequently are perhaps the first who need to acquire these new skills and to enhance their understanding of the care pathways, consent issues and utilisation of genomic knowledge in clinical management. However, it is expected that in the not-so-distant future, broader applications of genomic medicine such as pharmacogenetics and complex genetics will be increasingly important to all clinicians.

Technological advances of DNA sequencing technologies have tremendously expanded the ability of healthcare systems to diagnose RED. This gives great hope to affected families. Harnessing the motivating power of patient groups and hearing the patient voice is critical in promoting systematic change in healthcare provision. The ERN-EYE initiative has been strongly influenced by patient bodies and advocates. These interactions have greatly enhanced our understanding of how a definitive genetic diagnosis can promote closure, lead to early resolution of uncertainty, allow better understanding of the condition and, crucially, inform reproductive and life planning. However, ultimately, implementation of such advanced diagnostic strategies will require considerable increased investment. Thus, there is an urgent need for professionals to provide broad evidence of clinical benefit and utility. The extraordinary acceleration in the number of clinical trials for RED in general and for inherited retinal disorders in particular, has provided considerable urgency and impetus.

## Conclusions

- Technological advances have allowed genomic testing for RED.
- Despite these advances critical gaps in testing remain, especially in smaller countries where no formal genomic testing structures exist. Even within larger countries, the existing arrangements are insufficient to meet the demand and to ensure equity of access.
- The outcome of genetic testing allows better understanding of the condition and allows reproductive and therapeutic options. The increase of the number of clinical trials for RED has provided considerable urgency for genetic testing in RED.
- ERN-EYE promotes access to genetic testing in RED and emphasizes the clinical need and relevance of genetic testing in RED.

## Data Availability

The datasets used and/or analysed during the current study are available from the corresponding author on reasonable request.

## Abbreviations

ASO: Antisense oligonucleotide
Cas9: CRISPR associated protein 9
CRISPR: Clustered Regularly Interspaced Short Palindromic Repeats
EMA: European Medicines Agency
ERN: European Reference Network
ERN-EYE: European Reference Network for Rare Eye Diseases
FDA: Food and Drug Administration
LCA: Leber Congenital Amaurosis
IRD: Inherited Retinal Diseases
RED: Rare Eye Diseases
STGD1: Stargardt disease type 1

## References

[1] ERN description on European Commission official website [Internet]. Public Health - Eur. Comm. 2016 [cited 2020 Sep 29]. Available from: https://ec.europa.eu/health/ern_en

[2] ERN-EYE official website [Internet]. ERN-EYE. [cited 2020 Sep 29]. Available from: https://www.ern-eye.eu/

[3] Solebo AL, Teoh L, Rahi J (2017). Epidemiology of blindness in children. Arch Dis Child.

[4] Liew G, Michaelides M, Bunce C (2014). A comparison of the causes of blindness certifications in England and Wales in working age adults (16–64 years), 1999–2000 with 2009–2010. BMJ Open.

[5] Verbakel SK, van Huet RAC, Boon CJF, den Hollander AI, Collin RWJ, Klaver CCW (2018). Non-syndromic retinitis pigmentosa. Prog Retin Eye Res.

[6] Sergouniotis PI, Maxime E, Leroux D, Olry A, Thompson R, Rath A (2019). An ontological foundation for ocular phenotypes and rare eye diseases. Orphanet J Rare Dis.

[7] Zeggini E, Gloyn AL, Barton AC, Wain LV (2019). Translational genomics and precision medicine: Moving from the lab to the clinic. Science.

[8] Lenassi E, Clayton-Smith J, Douzgou S, Ramsden SC, Ingram S, Hall G (2020). Clinical utility of genetic testing in 201 preschool children with inherited eye disorders. Genet Med Off J Am Coll Med Genet.

[9] Claussnitzer M, Cho JH, Collins R, Cox NJ, Dermitzakis ET, Hurles ME (2020). A brief history of human disease genetics. Nature.

[10] Lasseaux E, Plaisant C, Michaud V, Pennamen P, Trimouille A, Gaston L (2018). Molecular characterization of a series of 990 index patients with albinism. Pigment Cell Melanoma Res.

[11] Kumaran N, Moore AT, Weleber RG, Michaelides M (2017). Leber congenital amaurosis/early-onset severe retinal dystrophy: clinical features, molecular genetics and therapeutic interventions. Br J Ophthalmol.

[12] Vázquez-Domínguez I, Garanto A, Collin RWJ. Molecular Therapies for Inherited Retinal Diseases-Current Standing, Opportunities and Challenges. Genes. 2019;10. 10.3390/genes10090654 PMC6770110 31466352

[13] Garafalo AV, Cideciyan AV, Héon E, Sheplock R, Pearson A, WeiYang YuC (2020). Progress in treating inherited retinal diseases: Early subretinal gene therapy clinical trials and candidates for future initiatives. Prog Retin Eye Res.

[14] Cideciyan AV, Jacobson SG (2019). Leber Congenital Amaurosis (LCA): Potential for Improvement of Vision. Invest Ophthalmol Vis Sci.

[15] Research C for BE and. Luxturna description on U.S Food and Drug website [Internet]. FDA. 2019 [cited 2020 Sep 26]. Available from: https://www.fda.gov/vaccines-blood-biologics/cellular-gene-therapy-products/luxturna

[16] Luxturna description on European Medicines Agency website [Internet]. EMA. 2018 [cited 2020 Sep 29]. Available from: https://www.ema.europa.eu/en/medicines/human/EPAR/luxturna

[17] Guidance for Luxturna on National Institute for Health and Care Excellence website [Internet]. NIH. NICE; [cited 2020 Sep 29]. Available from: https://www.nice.org.uk/guidance/hst11

[18] LUXTURNA on Haute Autorité de Santé website [Internet]. HAS. [cited 2020 Sep 29]. Available from: https://www.has-sante.fr/jcms/c_2964759/fr/luxturna

[19] Duijkers L, van den Born LI, Neidhardt J, Bax NM, Pierrache LHM, Klevering BJ, et al. Antisense Oligonucleotide-Based Splicing Correction in Individuals with Leber Congenital Amaurosis due to Compound Heterozygosity for the c.2991+1655A>G Mutation in CEP290. Int J Mol Sci. 2018;19. 10.3390/ijms19030753 PMC5877614 29518907

[20] Cideciyan AV, Jacobson SG, Drack AV, Ho AC, Charng J, Garafalo AV (2019). Effect of an intravitreal antisense oligonucleotide on vision in Leber congenital amaurosis due to a photoreceptor cilium defect. Nat Med.

[21] Ledford H (2020). CRISPR treatment inserted directly into the body for first time. Nature.

[22] Bachmann-Gagescu R, Neuhauss SC (2019). The photoreceptor cilium and its diseases. Curr Opin Genet Dev.

[23] Ellingford JM, Sergouniotis PI, Lennon R, Bhaskar S, Williams SG, Hillman KA (2015). Pinpointing clinical diagnosis through whole exome sequencing to direct patient care: a case of Senior-Loken syndrome. Lancet Lond Engl.

[24] Aboshiha J, Dubis AM, van der Spuy J, Nishiguchi KM, Cheeseman EW, Ayuso C (2015). Preserved outer retina in AIPL1 Leber’s congenital amaurosis: implications for gene therapy. Ophthalmology.

[25] Sacristan-Reviriego A, Le HM, Georgiou M, Meunier I, Bocquet B, Roux A-F (2020). Clinical and functional analyses of AIPL1 variants reveal mechanisms of pathogenicity linked to different forms of retinal degeneration. Sci Rep.

[26] Bouzia Z, Georgiou M, Hull S, Robson AG, Fujinami K, Rotsos T (2020). GUCY2D-associated leber congenital amaurosis: a retrospective natural history study in preparation for trials of novel therapies. Am J Ophthalmol.

[27] Jacobson SG, Cideciyan AV, Sumaroka A, Roman AJ, Charng J, Lu M (2017). Defining outcomes for clinical trials of leber congenital amaurosis caused by GUCY2D mutations. Am J Ophthalmol.

[28] Wright GA, Georgiou M, Robson AG, Ali N, Kalhoro A, Holthaus SK (2020). Juvenile batten disease (CLN3): detailed ocular phenotype, novel observations, delayed diagnosis, masquerades, and prospects for therapy. Ophthalmol Retina.

[29] Dulz S, Atiskova Y, Wibbeler E, Wildner J, Wagenfeld L, Schwering C (2020). An ophthalmic rating scale to assess ocular involvement in juvenile CLN3 disease. Am J Ophthalmol.

[30] Hirji N, Aboshiha J, Georgiou M, Bainbridge J, Michaelides M (2018). Achromatopsia: clinical features, molecular genetics, animal models and therapeutic options. Ophthalmic Genet.

[31] Cehajic Kapetanovic J, Patrício MI, MacLaren RE (2019). Progress in the development of novel therapies for choroideremia. Expert Rev Ophthalmol.

[32] De Silva SR, Arno G, Robson AG, Fakin A, Pontikos N, Mohamed MD, et al. The X-linked retinopathies: Physiological insights, pathogenic mechanisms, phenotypic features and novel therapies. Prog Retin Eye Res. 2020;100898. 10.1016/j.preteyeres.2020.100898 32860923

[33] Rahman N, Georgiou M, Khan KN, Michaelides M (2020). Macular dystrophies: clinical and imaging features, molecular genetics and therapeutic options. Br J Ophthalmol.

[34] Gill JS, Georgiou M, Kalitzeos A, Moore AT, Michaelides M. Progressive cone and cone-rod dystrophies: clinical features, molecular genetics and prospects for therapy. Br J Ophthalmol. 2019; 10.1136/bjophthalmol-2018-313278 PMC6709772 30679166

[35] Bauwens M, Garanto A, Sangermano R, Naessens S, Weisschuh N, De Zaeytijd J (2019). ABCA4-associated disease as a model for missing heritability in autosomal recessive disorders: novel noncoding splice, cis-regulatory, structural, and recurrent hypomorphic variants. Genet Med Off J Am Coll Med Genet.

[36] Sangermano R, Garanto A, Khan M, Runhart EH, Bauwens M, Bax NM (2019). Deep-intronic ABCA4 variants explain missing heritability in Stargardt disease and allow correction of splice defects by antisense oligonucleotides. Genet Med Off J Am Coll Med Genet.

[37] Khan M, Cornelis SS, Pozo-Valero MD, Whelan L, Runhart EH, Mishra K (2020). Resolving the dark matter of ABCA4 for 1054 Stargardt disease probands through integrated genomics and transcriptomics. Genet Med Off J Am Coll Med Genet.

[38] Shendure J, Balasubramanian S, Church GM, Gilbert W, Rogers J, Schloss JA (2017). DNA sequencing at 40: past, present and future. Nature.

[39] Boycott KM, Hartley T, Biesecker LG, Gibbs RA, Innes AM, Riess O (2019). A diagnosis for all rare genetic diseases: the horizon and the next frontiers. Cell.

[40] Lappalainen T, Scott AJ, Brandt M, Hall IM (2019). Genomic analysis in the age of human genome sequencing. Cell.

[41] Inherited retinal Disease Gene Testing Program on Invitae website [Internet]. INVITEAE. [cited 2020 Sep 29]. Available from: https://www.invitae.com/en/sponsored-testing/ophthalmology/

[42] Genetic Testing for Inherited Retinal Diseases through the Foundation’s Open Access Program on Fighting Blindness Foundation website [Internet]. Found. Fight. Blind. [cited 2020 Sep 29]. Available from: https://www.fightingblindness.org/research/genetic-testing-for-inherited-retinal-diseases-through-the-foundation-s-open-access-program-79

[43] Rare diseases description on official French government website [Internet]. DGOS Ministère Solidar. Santé. 2021 [cited 2020 Sep 29]. Available from: https://solidarites-sante.gouv.fr/soins-et-maladies/prises-en-charge-specialisees/maladies-rares/article/les-maladies-rares

[44] Plan France Médecine Génomique 2025 / aviesan [Internet]. [cited 2021 Jan 26]. Available from: https://www.aviesan.fr/aviesan/accueil/toute-l-actualite/plan-france-medecine-genomique-2025

[45] Genomics England website [Internet]. Genomics Engl. [cited 2020 Sep 29]. Available from: https://www.genomicsengland.co.uk/

[46] The Cost of Sequencing a Human Genome on National Human Genome Research Institute website [Internet]. [cited 2020 Sep 29]. Available from: https://www.genome.gov/about-genomics/fact-sheets/Sequencing-Human-Genome-cost

[47] Peterson JF, Roden DM, Orlando LA, Ramirez AH, Mensah GA, Williams MS (2019). Building evidence and measuring clinical outcomes for genomic medicine. Lancet Lond Engl.

[48] Sergouniotis PI (2019). Inherited retinal disorders: using evidence as a driver for implementation. Ophthalmol J Int Ophtalmol Int J Ophthalmol Z Augenheilkd.

[49] Gillespie RL, Urquhart J, Anderson B, Williams S, Waller S, Ashworth J (2016). Next-generation sequencing in the diagnosis of metabolic disease marked by pediatric cataract. Ophthalmology.

[50] Gillespie RL, O’Sullivan J, Ashworth J, Bhaskar S, Williams S, Biswas S, et al. Personalized diagnosis and management of congenital cataract by next-generation sequencing. Ophthalmology. 2014;121:2124–2137.e1–2. 10.1016/j.ophtha.2014.06.006 25148791

[51] Taylor RL, Parry NRA, Barton SJ, Campbell C, Delaney CM, Ellingford JM (2017). Panel-based clinical genetic testing in 85 children with inherited retinal disease. Ophthalmology.

[52] Haer-Wigman L, van Zelst-Stams WA, Pfundt R, van den Born LI, Klaver CC, Verheij JB (2017). Diagnostic exome sequencing in 266 Dutch patients with visual impairment. Eur J Hum Genet EJHG.

[53] Birtel J, Gliem M, Oishi A, Müller PL, Herrmann P, Holz FG (2019). Genetic testing in patients with retinitis pigmentosa: Features of unsolved cases. Clin Experiment Ophthalmol.

[54] Carss KJ, Arno G, Erwood M, Stephens J, Sanchis-Juan A, Hull S (2017). Comprehensive rare variant analysis via whole-genome sequencing to determine the molecular pathology of inherited retinal disease. Am J Hum Genet.

